# Supplementary figures and images for: Parascedosporium putredinis NO1 tailors its secretome for different lignocellulosic substrates
Source: Microbiol Spectr. 2024 May 17;12(7):e03943-23. doi: 10.1128/spectrum.03943-23 (PMC11218486; doi:10.1128/spectrum.03943-23)

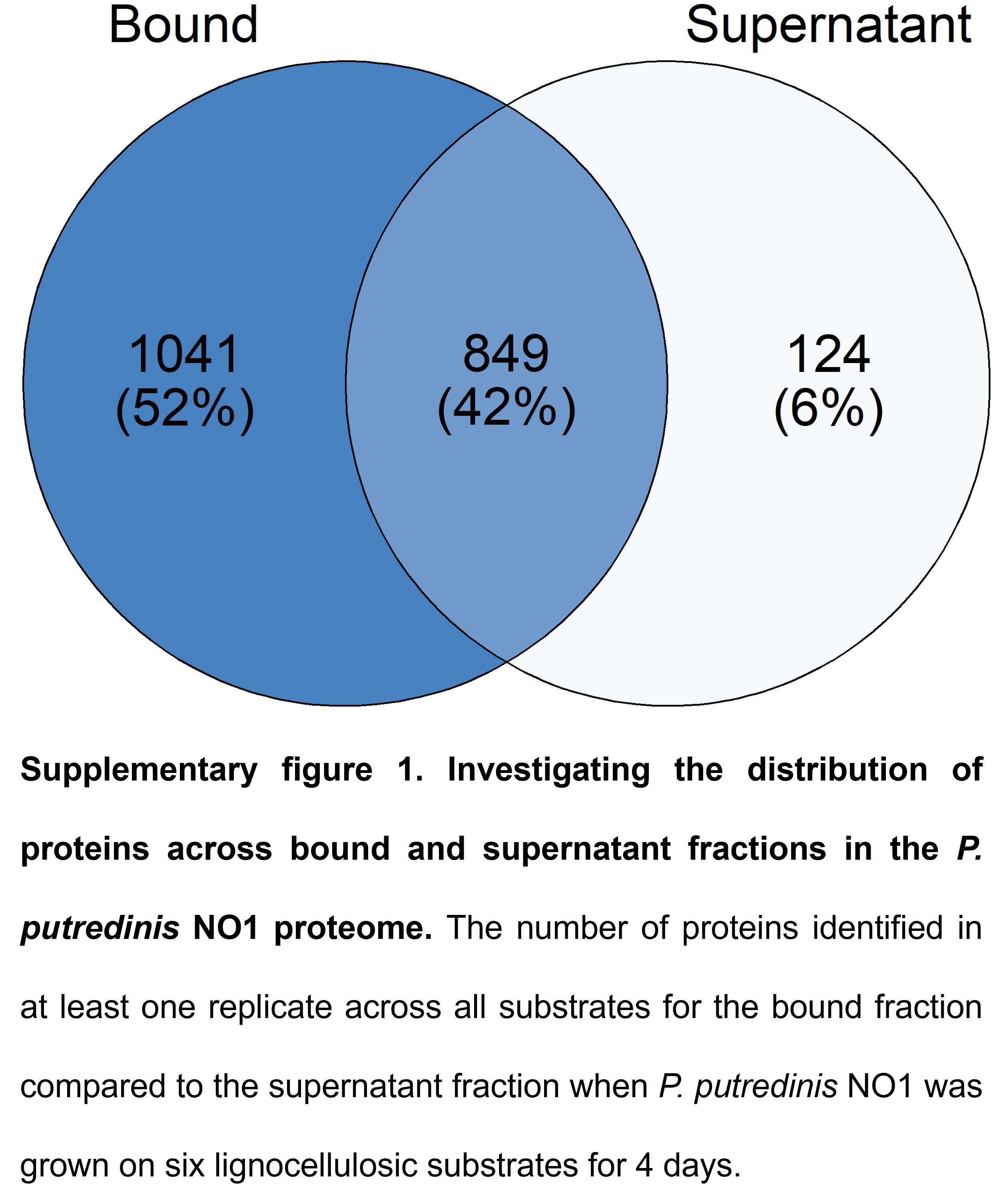

Supplement: Figure S1 — Investigating the distribution of protein. [file spectrum.03943-23-s0001.tiff]

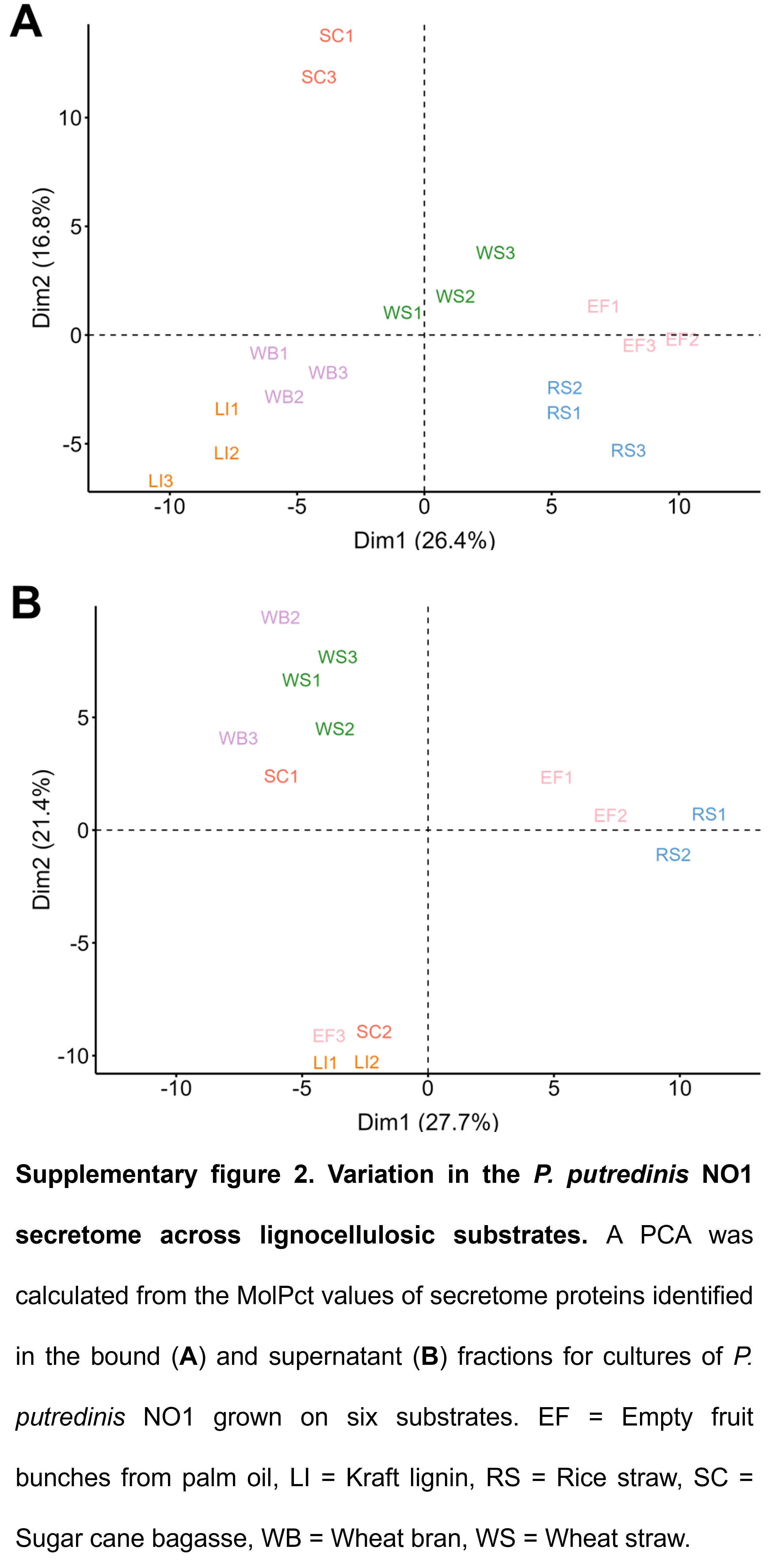

Supplement: Figure S2 — Variation in the P. putredinis NO1 secretome. [file spectrum.03943-23-s0002.tiff]

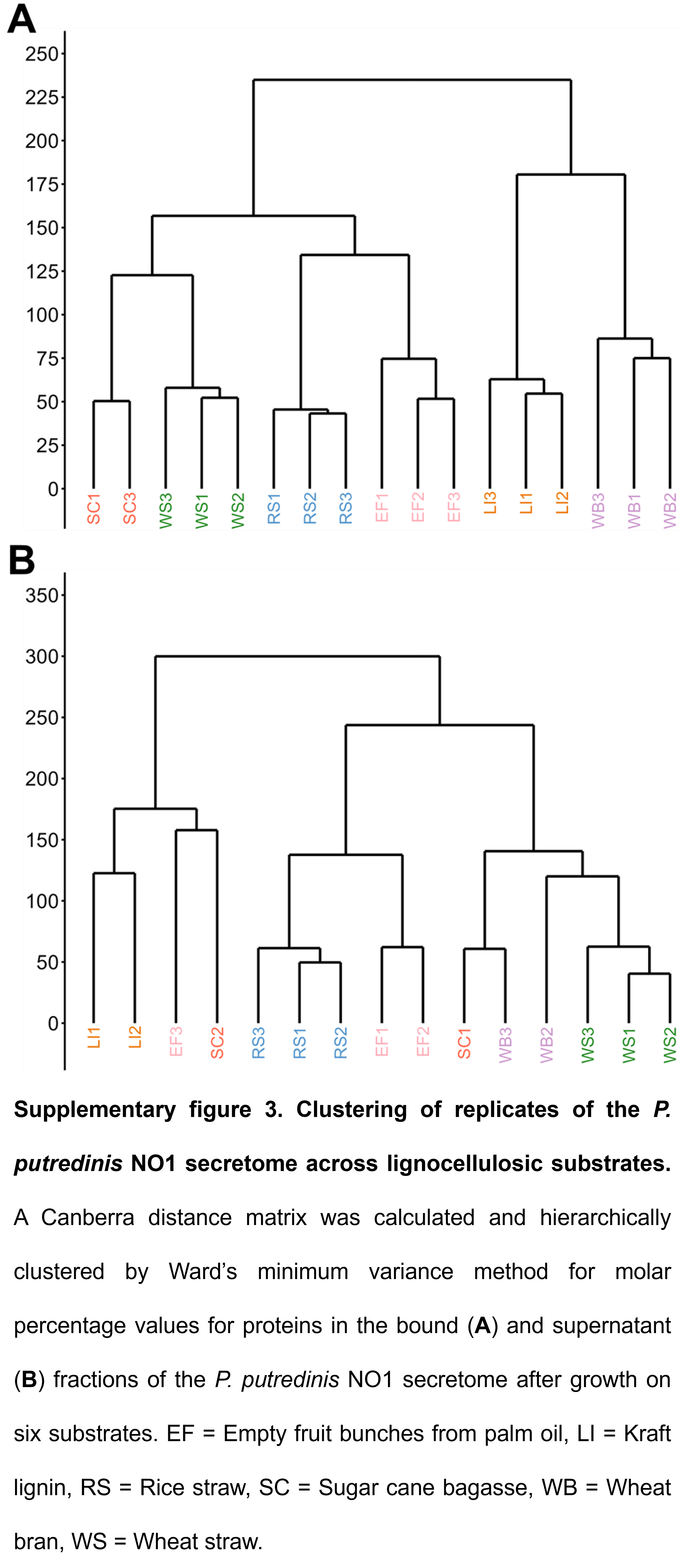

Supplement: Figure S3 — Clustering of replicates of the P. putredinis NO1 secretome. [file spectrum.03943-23-s0003.tiff]

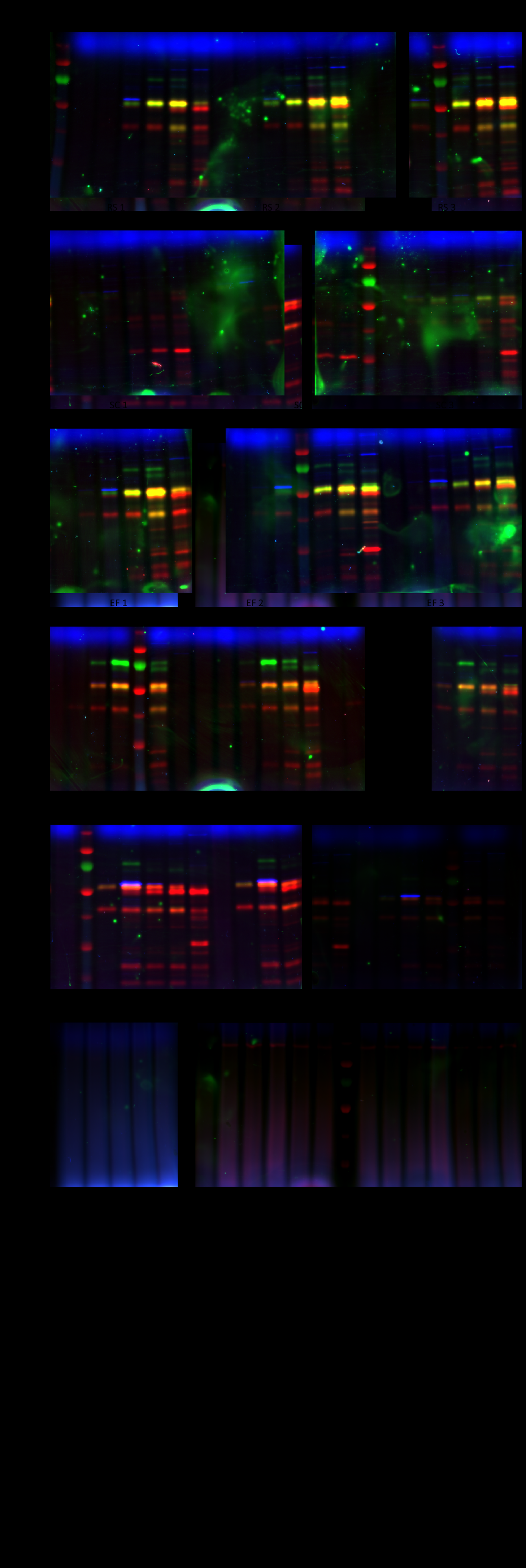

Supplement: Figure S4 — Differences in P. putredinis NO1 glycoside hydrolase production. [file spectrum.03943-23-s0004.tiff]
